# Adherence to wearing prescribed footwear in people at risk of diabetes‐related foot ulcers

**DOI:** 10.1002/jfa2.70002

**Published:** 2024-08-25

**Authors:** Gustav Jarl, Chantal M. Hulshof, Kim A. Tijhuis, Tessa E. Busch‐Westbroek, Sicco A. Bus, Jaap J. van Netten

**Affiliations:** ^1^ Department of Prosthetics and Orthotics Faculty of Medicine and Health Örebro University Örebro Sweden; ^2^ University Health Care Research Center Faculty of Medicine and Health Örebro University Örebro Sweden; ^3^ Department of Rehabilitation Medicine Amsterdam UMC Location University of Amsterdam Amsterdam The Netherlands; ^4^ Amsterdam Movement Sciences, Rehabilitation & Development Amsterdam The Netherlands

**Keywords:** diabetic foot, foot ulcer, patient compliance, prevention, shoes

## Abstract

**Introduction:**

Adherence to wearing prescribed footwear is paramount in reducing the risk of developing diabetes‐related foot ulcers, but adherence is often lower than optimal. This study aimed to investigate predictors of footwear adherence and variations in adherence and activity in people at risk of diabetes‐related foot ulceration.

**Methods:**

Sixty people at high foot ulcer risk were included. We measured the proportion of weight‐bearing acitivity time the prescribed footwear was worn for seven days. Multiple linear regression and analysis of variance were used.

**Results:**

Mean overall adherence was 63%. Adherence was lower at home than away from home (59% vs. 74%), while activity was higher at home (2.2 vs. 1.2 h/day). Adherence was similar across activities (61%–63%). No variable predicted the overall adherence. Higher Hba1c predicted lower adherence at home (*β* = −0.34, *p* = 0.045, *R* ^2^ = 11.6%). More daily steps predicted lower adherence away from home (*β* = −0.30, *p* = 0.033, *R* ^2^ = 9.3%). Adherence and activity were highest in mornings (71%, 1.1 h) and afternoons (71%, 1.5 h), and lower in evenings (40%, 0.8 h) and at nights (9%, 0.1 h). Adherence was similar on weekdays and weekend days (63% vs. 60%), but activity was higher on weekdays (3.4 vs. 3.0 h).

**Conclusion:**

Adherence levels and predictors thereof differed between adherence at home and away from home, so we suggest to treat them as different concepts. Due to the low explained variance, future studies should focus on other predictors such as psychological variables.

## 1 INTRODUCTION

Adherence to wearing prescribed footwear is paramount in reducing the risk of developing diabetes‐related foot ulcers [1, 2]. Foot ulcers are a common and devastating long‐term complication of diabetes, affecting approximately 19%–34% of people with diabetes during their life time [3]. Foot ulcers are associated with low quality of life, short life expectancy, risk of lower limb amputation, and high health care costs [3]. In addition, recurrence rates of foot ulcers are alarmingly high, approximately 40% within 1 year [3]. Hence, reducing the risk of first‐time and recurrent foot ulcers is of utmost importance. International guidelines recommend using therapeutic footwear for reducing the risk of foot ulcer [1]. However, a recent systematic review found that only 28%–60% of the participants wore their prescribed footwear for more than 80% of their daily steps or the day [4]. To improve adherence and help prevent foot ulcers, we need to better understand the factors contributing to low adherence in order to develop and implement interventions that address these factors.

Only three studies using objective adherence measurements and seven studies using subjective measurements investigated predictors of wearing footwear to prevent diabetes‐related foot ulcers [4, 5, 6, 7, 8]. However, subjective measures of adherence are found to be unreliable [9]. The three studies using objective measurements found no significant difference between women and men [5, 6, 7]. One study [5] found that lower BMI, more severe foot deformity, and more esthetically appealing footwear were associated with higher adherence, while another study [7] found that higher self‐reported satisfaction with wearing time of footwear was associated with higher objective wearing time of footwear. Thus, results on what factors predict footwear adherence are inconclusive, and both studies had low explained variance in multivariate models. An explanation for this may be that most studies have focused on overall adherence. That is, in most studies weight‐bearing activity duration and adherence to footwear have been treated as a constant phenomenon, without considering variations across times, locations, and activities. However, studies have found that weight‐bearing activity is higher on weekdays compared to weekend days, and higher at home compared to away from home; moreover, they also found that adherence is lower at home compared to away from home [5, 10, 11]. These results suggest that weight‐bearing activity and adherence to wearing prescribed footwear vary and predictors may need to be investigated specifically for different times (over the day and week), locations (home vs. away from home), and activities.

The aim of this study was to investigate predictors of adherence to wearing prescribed footwear and variations in adherence and the level of activity over the day, week, locations (home and away from home) and activities in people with diabetes who are at high risk of foot ulceration.

## 2 MATERIALS AND METHODS

### 2.1 Ethics

The study conformed to the Declaration of Helsinki. The local medical ethics committee of Amsterdam UMC ruled the study exempt from the requirement for ethical review according to the Dutch law under the Medical Research Involving Human Subjects Act in the Netherlands (registration number: W19_429#19.495). All participants provided written informed consent prior to study participation.

### 2.2 Participants

Participants were recruited as part of the prospective observational cohort study DIALOAD (https://www.trialregister.nl/trial/8839) at two locations of Amsterdam UMC and podiatry practice Voeten op Texel, all located in the Netherlands. A total of 103 potential participants were identified at their regular clinical appointment, informed about the study, and asked for willingness to participate after a week. In total, 63 people agreed to participate of which 3 did not meet the eligibility criteria, resulting in 60 participants.

Inclusion criteria were age ≥18 years, being ambulatory, diabetes type 1 or 2, loss of protective sensation (inability to feel a 10‐g monofilament and tuning fork following criteria of the IWGDF guidelines [12]) and a recent history of a foot ulcer (<1 year) or high dynamic forefoot/midfoot barefoot peak plantar pressure (>600 kPa). Exclusion criteria were use of a walking aid for full body support (e.g., wheelchair), severe peripheral artery disease (WIfI grade 3 [13]), foot ulcer, open amputation site, and active Charcot neuro‐osteoarthropathy.

### 2.3 Procedures

At baseline, participants underwent a physical examination, performed tests, and answered questionnaires. The baseline data were collected with validated methods and used as potential predictors of adherence in the regression analyses. Mobility and proactive balance were assessed with the Timed Up and Go (TUG) test, in which the time was recorded to raise from a chair, walk to a cone 3 m away, turn, walk back, and sit down again. Function of the lower extremities was assessed with the short physical performance battery (SPPB), which includes three aspects: balance, gait speed, and rise from a chair. The SPPB produces a score between 0 and 12, where a higher score reflects better function. We included TUG and SPPB as they are associated with higher adherence to exercise programs, and to explore if physical capacity parameters might be associated with adherence to footwear. Dynamic barefoot plantar pressures were measured four times per foot according to the 2‐step protocol using the EMED platform (Novel GmbH) [14]. In‐shoe plantar pressures during walking were measured with the Pedar‐X system (Novel GmbH) collecting data for at least 12 mid‐gait steps [15]. Peak pressure was defined as the highest measured pressure during the whole stance phase. The average peak plantar pressure was defined as the sum of the peak pressures for each valid step, divided by the number of valid steps. Two questionnaires were completed: the Short Form 36 (SF‐36) and the Monitor Orthopaedic Shoes questionnaire for participants with semi custom‐made footwear (prefabricated shoes with custom‐made insoles) or fully custom‐made footwear [1].

To assess weight‐bearing activities, participants wore a tri‐axial accelerometer (DynaPort MoveMonitor, McRoberts) for seven consecutive days after the baseline visit [16]. Participants were instructed to wear the accelerometer in the middle of their back, at level L5, and only remove it during water activities. The accelerometer has a 100 Hz sampling frequency, 12‐bit resolution, and a range of ±6 g. The accelerometer had to be worn ≥75% of 24 h [17], or ≥12 h if not worn at night, to count as a valid day [18]. Wearing time of the footwear was assessed during the same 7 days the accelerometer was worn, using a validated temperature sensor to determine when the footwear was worn or not worn (Orthotimer, Rollerwerk) [19]. The sensor was secured in up to four pairs of prescribed footwear (invisible in the insole's medial arch support underneath the top layer) of each participant [19]. The sensor measured and stored time‐stamped temperatures every 15 min. For each participant, at least four valid days of both activity and temperature data were required for analysis, with a day considered valid if it included both valid activity and wearing time data [17]. For participants with less than four valid days, we used multiple imputation (see below) to ensure at least four valid days for every participant. For comparisons of adherence and activity between weekdays and weekend days, valid data were required for at least 2 week days and two weekend days. In addition, participants completed a daily logbook, noting when they were at home and away from home.

### 2.4 Analysis

We used multiple imputation using a fully conditional specific predictive mean matching model for missing data on activity and adherence for participants with less than four valid days of data. Imputations were generated by drawing from iterated conditional models, using variables associated with the missing data as predictors. Adherence was defined as objectively measured proportion of weight‐bearing activity time the prescribed footwear was worn. We used the manufacturer's validated algorithm to categorize the accelerometer raw data into periods of walking, standing, shuffling, stair walking, lying, sitting, cycling, and non‐wearing [20]. Walking, standing, shuffling and stair walking were defined as weight‐bearing activities. The average number of daily steps and daily weight‐bearing duration over the day, week, locations (home and away from home), and activities of walking and stair walking were determined using custom‐written MATLAB scripts (R2021b, the MathWorks, Inc.). The raw data from the temperature sensors were analyzed with the Groningen algorithm, version 2 (https://github.com/CHulshof/Orthotimer_algorithm) to determine when the footwear was worn and not worn [21]. Finally, adherence to wearing footwear was determined by combining weight‐bearing activity and wearing time data using custom‐made MATLAB scripts and averaged over valid days.

Potential predictors of adherence were analyzed separately for overall adherence, adherence at home, and adherence away from home. First, a linear regression analysis was performed between each potential predictor and adherence. Predictors with a *p*‐value <0.2 were entered into a backward multiple linear regression analysis. Collinearity between predictors was investigated by calculating the variance inflation factor (VIF) and investigating Spearman's correlation coefficient between the predictors. When substantial collinearity was detected (a VIF >2.5 [22], or a correlation coefficient >0.7 or <−0.7), we excluded the predictor having weaker association with the dependent variable and reran the regression analysis. *R* ^2^ was calculated as a measure of the proportion of variance that the final regression model explained. We calculated Pearson's correlation coefficient between overall adherence, adherence at home, and adherence away from home. We used repeated measures one‐way analysis of variance (ANOVA) to compare activity duration, number of daily steps, and adherence levels across the day, week, locations, and activities. We used IBM SPSS Statistics 28 for Windows (Armonk, NY: IBM Corp.) for statistical analyses.

## 3 RESULTS

Of the 60 participants, 49 were men and 11 were women with a mean age of 65.3 years. The majority of the participants had type 2 diabetes (82%) and a history of foot ulceration (97%) (Table 1).

**TABLE 1:** Predictors of overall footwear adherence (*n* = 60 with missing data on some variables).

	*N* (%) or mean (SD)	Mean adherence (SD)	Univariate regression		Multiple regression	
			*β*	*p*‐value	*β*	*p*‐value
*Demographic and clinical variables*						
Sex						
Woman	11 (18)	58 (28)	−0.12	0.35		
Man (ref)	49 (82)	65 (20)				
Age	65.3 (9.2)	‐‐‐	−0.06	0.65		
Education level						
Low (ref)	16 (27)	57 (29)		0.42^b^		
Medium	15 (25)	66 (18)	0.17			
High	29 (48)	65 (18)	0.19			
Work situation						
Employed	26 (43)	63 (17)	−0.02	0.87		
Not employed (ref)	34 (57)	64 (25)				
Living alone						
Yes	27 (45)	65 (19)	0.06	0.66		
No (ref)	33 (55)	62 (23)				
Mobility device						
Yes	14 (23)	65 (22)	0.05	0.69		
No (ref)	46 (77)	63 (21)				
Daily steps	5633 (3575)	‐‐‐	−0.14	0.28		
Variation in daily steps^a^	1925 (1402)	‐‐‐	−0.01	0.95		
Short physical performance battery	8.6 (2.9)	‐‐‐	−0.25	0.09^c^		NS
Timed up and go test, seconds	13.4 (3.7)	‐‐‐	0.18	0.17		
In‐shoe mid‐gait mean peak pressure, kPa	197.3 (73.8)	‐‐‐	−0.10	0.46		
Barefoot mid‐gait mean peak pressure, kPa	1038 (213)	‐‐‐	0.08	0.52		
Body mass index	29.6 (5.4)	‐‐‐	−0.05	0.73		
Diabetes type						
Type 1 (ref)	11 (18)	64 (26)				
Type 2	49 (82)	63 (20)	−0.01	0.95		
Diabetes duration, years	18.8 (11.5)	‐‐‐	−0.05	0.68		
Hba1c	60.3 (17.4)	‐‐‐	−0.24	0.09^c^		NS
Time since foot ulcer healing, months	12.8 (29.7)	‐‐‐	−0.05	0.74		
Current smoker						
Yes	5 (8)	62 (26)	−0.03	0.84		
No (ref)	55 (92)	63 (21)				
Alcohol use						
Yes	40 (67)	64 (20)	0.04	0.77		
No (ref)	20 (33)	62 (25)				
Charcot foot deformity						
Yes	7 (12)	64 (20)	0.01	0.92		
No (ref)	53 (88)	63 (22)				
Amputation						
Yes	23 (38)	63 (22)	−0.03	0.85		
No (ref)	37 (62)	64 (21)				
Footwear type						
Conventional shoes, custom‐made insoles	7 (12)	64 (14)	0.04			
Prefabricated shoes, custom‐made insoles	15 (25)	68 (22)	0.14			
Fully custom‐made footwear (ref)	38 (63)	61 (22)		0.56^b^		
Owns prescribed indoor footwear						
Yes	12 (20)	59 (27)	−0.11	0.41		
No (ref)	48 (80)	64 (20)				
Number of prescribed pairs of footwear						
1–2 pairs (ref)	25 (42)	62 (21)				
≥3 pairs	35 (58)	64 (21)	0.07	0.62		
*Footwear usability (MOS questionnaire), score 0–100 if not otherwise stated*						
Walking ability^d^						
0–10 m/10–50 m/50–200 m	12 (23)	63 (27)	0.00			
200–1000 m	15 (29)	63 (20)	0.00			
>1000 m (ref)	25 (48)	63 (23)		1.00^b^		
Pain in skin of feet	20 (25)	‐‐‐	−0.09	0.52		
Pain in muscles and joints of feet	26 (28)	‐‐‐	−0.10	0.48		
Aesthetics of footwear according to self	58 (28)	‐‐‐	0.19	0.18^c^		NS
Aesthetics of footwear according to others^d^						
Ugly/Very ugly	6 (15)	64 (17)	−0.03			
Neutral	14 (34)	71 (24)	0.13			
Attractive/Very attractive (ref)	21 (51)	65 (17)		0.67^b^		
Fit of footwear	80 (17)	‐‐‐	0.26	0.06^c^		NS
Walking ability with footwear	79 (22)	‐‐‐	0.20	0.16		
Ease of donning/doffing footwear	68 (24)	‐‐‐	0.17	0.22		
Satisfied with wearing time of footwear	75 (20)	‐‐‐	0.01	0.96		
Physician listened to me	85 (14)	‐‐‐	0.16	0.25		
Shoe technician listened to me	88 (13)	‐‐‐	0.01	0.94		
Prioritizes footwear solves foot problems over aesthetics	79 (18)	‐‐‐	0.06	0.65		
Advantages of footwear outweigh disadvantages	77 (24)	‐‐‐	0.02	0.89		
*Quality of life (SF–36), score 0–100*						
Physical functioning	63 (24)	‐‐‐	−0.17	0.20		
Role—physical	55 (42)	‐‐‐	−0.02	0.89		
Bodily pain	73 (24)	‐‐‐	0.13	0.32		
General health	49 (21)	‐‐‐	−0.09	0.48		
Vitality	59 (21)	‐‐‐	0.00	0.99		
Social functioning	78 (25)	‐‐‐	0.01	0.93		
Role—emotional	78 (34)	‐‐‐	−0.07	0.60		
Mental health	75 (17)	‐‐‐	0.08	0.56		

Abbreviations: MOS, Monitor Orthopedisch Schoeisel; NS, not significant (*p* > 0.05); SD, standard deviation.

^a^ Calculated as the standard deviation of the number of steps per valid day.

^b^ For categorical variables with more than two categories, the overall *p*‐value for the variable was calculated.

^c^ Included in multiple regression analysis.

^d^ Response categories were combined due to few answers in the categories.

Mean overall adherence was 63% (SD:21%), meaning that participants used their prescribed footwear for 63% of weight‐bearing activity time. Six variables had *p*‐values <0.2 (Table 1). We stepwise excluded two variables due to high correlation coefficient: TUG score (*r* = −0.72 with SPPB score) and ‘Walking ability with footwear’ (*r* = 0.80 with ‘Fit of footwear’). The remaining four variables, SPPB score, HbA1c, ‘Aesthetics of footwear according to self’, and ‘Fit of footwear’, had acceptable correlations (from −0.28 to 0.58) and VIF (from 1.0 to 1.4). In the multiple regression analysis, HbA1c was the last remaining variable in the model, but did not reach statistical significance (*β* = −0.31, *p* = 0.06).

Mean adherence at home was 59% (SD:21%). Eight variables had *p*‐values <0.2 (Table 2). We stepwise excluded two variables due to high correlation coefficient: TUG score (*r* = −0.72 with SPPB score), and ‘Walking ability with footwear’ (*r* = 0.80 with ‘Fit of footwear’). The remaining six variables, number of daily steps, SPPB score, Hba1c, ‘Fit of footwear’, ‘Ease of donning/doffing footwear’, and ‘Physician listened’ had acceptable correlations (from −0.40 to 0.64) and VIF (from 1.2 to 1.9). In the multiple regression, higher HbA1c was associated with lower footwear adherence at home (*β* = −0.34, *p* = 0.045) and the model explained 11.6% of the variance in adherence at home (*R* ^2^ = 0.116, adjusted *R* ^2^ = 0.089).

**TABLE 2:** Predictors of footwear adherence at home and away from home (*n* = 60 with missing data on some variables).

	Footwear adherence at home					Footwear adherence away from home				
	Mean adherence (SD)	Univariate regression		Multiple regression		Mean adherence (SD)	Univariate regression		Multiple regression	
		*β*	*p*‐value	*β*	*p*‐value		*β*	*p*‐value	*β*	*p*‐value
*Demographic and clinical variables*										
Sex										
Woman	56 (28)	−0.07	0.58			73 (32)	−0.03	0.84		
Man (ref)	60 (19)					74 (23)				
Age	‐‐‐	0.00	1.00			‐‐‐	0.00	0.99		
Education level										
Low (ref)	53 (27)		0.41^b^			71 (32)		0.82^b^		
Medium	61 (18)	0.16				76 (24)	0.10			
High	62 (18)	0.21				75 (22)	0.08			
Work situation										
Employed	58 (16)	−0.07	0.58			70 (21)	−0.15	0.28		
Not employed (ref)	61 (24)					77 (27)				
Living alone										
Yes	60 (19)	0.01	0.92			79 (21)	0.18	0.16^c^		NS
No (ref)	59 (23)					70 (27)				
Mobility device										
Yes	62 (23)	0.08	0.52			85 (27)	0.25	0.06^c^		NS
No (ref)	58 (21)					71 (24)				
Number of daily steps	‐‐‐	−0.21	0.10^c^		NS	‐‐‐	−0.33	0.01^c^	−0.30	0.03
Variation in daily steps^a^	‐‐‐	−0.13	0.37			‐‐‐	−0.11	0.43		
Short physical performance battery	‐‐‐	−0.31	0.04^c^		NS	‐‐‐	−0.36	0.01		
Timed up and go test, seconds	‐‐‐	0.25	0.06			‐‐‐	0.25	0.06		
In‐shoe mid‐gait mean peak pressure, kPa	‐‐‐	−0.15	0.24			‐‐‐	−0.02	0.88		
Barefoot mid‐gait mean peak pressure, kPa	‐‐‐	0.11	0.42			‐‐‐	0.23	0.08^c^		NS
Body mass index	‐‐‐	−0.08	0.57			‐‐‐	0.06	0.66		
Diabetes type										
Type 1 (ref)	58 (23)					73 (27)				
Type 2	60 (21)	0.03	0.82			74 (25)	0.03	0.84		
Diabetes duration, years	‐‐‐	−0.06	0.66			‐‐‐	−0.03	0.83		
Hba1c	‐‐‐	−0.30	0.03^c^	−0.34	0.045	‐‐‐	−0.04	0.79		
Time since foot ulcer healing, months	‐‐‐	−0.04	0.75			‐‐‐	−0.01	0.96		
Current smoker										
Yes	56 (25)	−0.05	0.73			71 (28)	−0.03	0.81		
No (ref)	60 (21)					74 (25)				
Alcohol use										
Yes	60 (19)	0.06	0.65			74 (23)	−0.02	0.86		
No (ref)	58 (25)					75 (29)				
Charcot foot deformity										
Yes	62 (20)	0.04	0.74			80 (24)	0.09	0.50		
No (ref)	59 (21)					73 (25)				
Amputation										
Yes	61 (22)	0.05	0.73			72 (27)	−0.06	0.65		
No (ref)	59 (21)					75 (24)				
Footwear type										
Conventional shoes, custom‐made insoles	57 (13)	−0.03				69 (17)	−0.07			
Prefabricated shoes, custom‐made insoles	62 (23)	0.07				77 (21)	0.05			
Fully custom‐made footwear (ref)	59 (22)		0.82^b^			74 (28)		0.80^b^		
Owns prescribed indoor footwear										
Yes	58 (26)	−0.04	0.74			66 (34)	−0.17	0.20		
No (ref)	60 (20)					76 (22)				
Number of prescribed pairs of footwear										
1–2 pairs (ref)	57 (20)					75 (23)				
≥3 pairs	61 (22)	0.09	0.48			73 (27)	−0.03	0.84		
*Footwear usability (MOS questionnaire), score 0–100 if not otherwise stated*										
Walking ability^d^										
0–10 m/10–50 m/50–200 m	61 (26)	0.04				81 (31)	0.21			
200–1000 m	62 (22)	0.08				79 (19)	0.20			
>1000 m (ref)	58 (21)		0.88^b^			68 (27)		0.24^b^		
Pain in skin of feet	‐‐‐	−0.05	0.75			‐‐‐	−0.03	0.83		
Pain in muscles and joints of feet	‐‐‐	−0.08	0.58			‐‐‐	−0.02	0.92		
Aesthetics of footwear according to self	‐‐‐	0.18	0.21			‐‐‐	0.13	0.34		
Aesthetics of footwear according to others^d^										
Ugly/Very ugly	62 (16)	0.02				74 (29)	−0.08			
Neutral	67 (21)	0.16				79 (29)	−0.01			
Attractive/Very attractive (ref)	61 (19)		0.64^b^			80 (16)		0.89^b^		
Fit of footwear	‐‐‐	0.24	0.09^c^		NS	‐‐‐	0.08	0.57		
Walking ability with footwear	‐‐‐	0.19	0.17			‐‐‐	−0.04	0.80		
Ease of donning/doffing footwear	‐‐‐	0.19	0.17^c^		NS	‐‐‐	0.07	0.64		
Satisfied with wearing time of footwear	‐‐‐	0.00	0.97			‐‐‐	0.19	0.17^c^		NS
Physician listened to me	‐‐‐	0.20	0.17^c^		NS	‐‐‐	0.13	0.36		
Shoe technician listened to me	‐‐‐	0.06	0.70			‐‐‐	0.05	0.72		
Prioritizes footwear solves foot problems over aesthetics	‐‐‐	−0.02	0.88			‐‐‐	0.17	0.24		
Advantages of footwear outweigh disadvantages	‐‐‐	0.09	0.54			‐‐‐	0.08	0.57		
*Quality of life (SF–36), score 0–100*										
Physical functioning	‐‐‐	−0.17	0.21			‐‐‐	−0.44	<0.001		
Role—physical	‐‐‐	−0.02	0.90			‐‐‐	−0.19	0.18^c^		NS
Bodily pain	‐‐‐	0.12	0.36			‐‐‐	0.03	0.84		
General health	‐‐‐	−0.10	0.46			‐‐‐	−0.06	0.66		
Vitality	‐‐‐	−0.04	0.74			‐‐‐	−0.12	0.36		
Social functioning	‐‐‐	−0.03	0.85			‐‐‐	−0.04	0.78		
Role—emotional	‐‐‐	−0.12	0.38			‐‐‐	−0.19	0.16^c^		NS
Mental health	‐‐‐	0.00	1.00			‐‐‐	0.11	0.41		

Abbreviations: MOS, Monitor Orthopedisch Schoeisel; SD, standard deviation.

^a^ Calculated as the standard deviation of the number of steps per valid day.

^b^ For categorical variables with more than two categories, the overall *p*‐value for the variable was calculated.

^c^ Included in multiple regression analysis.

^d^ Response categories were combined due to few answers in the categories.

Mean adherence away from home was 74% (SD: 25%). Ten variables had *p*‐values <0.2 (Table 2). We stepwise excluded three variables due to high correlation coefficient and/or high VIF: TUG score (*r* = −0.72 with SPPB score), SPPB score (*r* = −0.70 with mobility device, VIF = 5.7), and physical functioning (VIF = 2.9). The remaining seven variables, living alone, mobility device, number of daily steps, barefoot mid‐gait mean peak pressure, ‘Satisfied with wearing time of footwear’, Role‐physical, and Role‐emotional had acceptable correlations (from −0.40 to 0.38) and VIF (from 1.1 to 1.5). In multiple regression, higher number of daily steps was associated with lower footwear adherence away from home (*β* = −0.30, *p* = 0.033) and the model explained 9.3% of the variance of adherence away from home (*R* ^2^ = 0.093, adjusted *R* ^2^ = 0.073).

The correlation coefficient was 0.94 between overall adherence and adherence at home, 0.78 between overall adherence and adherence away from home, and 0.67 between adherence at home and adherence away from home.

**FIGURE 1:**
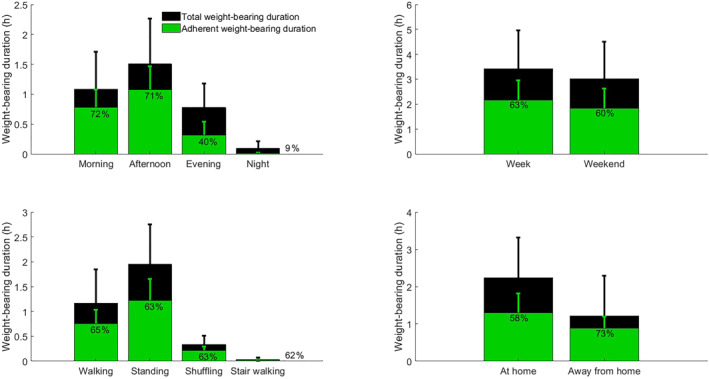
Variations of weight‐bearing activity and adherence to prescribed footwear.

Participants spent on average 3.5 h/day in weight‐bearing activities. Adherence, weight‐bearing activity duration, and number of steps followed a similar pattern over the day and ranked from highest to lowest: afternoons (71% adherence, 1.5 h activity, 2748 steps), mornings (71%, 1.1 h, 1879 steps), evenings (40%, 0.8 h, 1067 steps), and nights (9%, 0.1 h, 112 steps) (Table 3, Figure 1). All differences were statistically significant, except for adherence in the morning versus in the afternoon.

**TABLE 3:** Differences in activity duration, daily steps, and adherence over the day, week, locations, and activities.

	Mean adherence, % (SD)	Comparisons of adherence^a^ ^,^ ^b^	Mean weight‐bearing activity, h/day (SD)	Comparisons of weight‐bearing activity^a^ ^,^ ^b^	Mean number of daily steps (SD)	Comparisons of number of daily steps^a^ ^,^ ^b^
Overall	63 (21)	—	3.5 (1.5)	—	5633 (3575)	—
Over the day		F_(3,48)_ = 77.647 (*p* < 0.001)		F_(3,50)_ = 78.883 (*p* < 0.001)		F_(3,50)_ = 42.646 (*p* < 0.001)
Morning (6–12a.m.)	71 (28)	a	1.1 (0.6)	a	1879 (1784)	a
Afternoon (12a.m.–6p.m.)	71 (26)	a	1.5 (0.8)	b	2748 (1942)	b
Evening (6–12p.m.)	40 (30)	b	0.8 (0.4)	c	1067 (821)	c
Night (12p.m.–6a.m.)	9 (19)	c	0.1 (0.1)	d	112 (207)	d
Over the week		F_(1,52)_ = 0.896 (*p* = 0.35)		F_(1,52)_ = 7.210 (*p* = 0.01)		F_(1,52)_ = 6.392 (*p* = 0.015)
Weekdays (Mon–Fri)	63 (23)	a	3.4 (1.5)	a	5782 (3727)	a
Weekend days (sat–Sun)	60 (27)	a	3.0 (1.5)	b	5036 (3849)	b
Over locations		F_(1,59)_ = 34.867 (*p* < 0.001)		F_(1,47)_ = 21.312 (*p* < 0.001)		F_(1,47)_ = 0.022 (*p* = 0.88)
At home	59 (21)	a	2.2 (1.1)	a	2866 (2138)	a
Away from home	74 (25)	b	1.2 (1.1)	b	2930 (2732)	a
Over activities		F_(3,47)_ = 1.223 (*p* = 0.31)		F_(3,50)_ = 98.702 (*p* < 0.001)		F_(1,52)_ = 128.899 (*p* < 0.001)
Walking	63 (23)	a	1.2 (0.7)	a	5702 (3649)	a
Standing	61 (22)	a	2.0 (0.8)	b	NA	NA
Shuffling	61 (22)	a	0.3 (0.2)	c	NA	NA
Stair walking	62 (26)	a	0.03 (0.03)	d	133 (136)	b

*Note*: The same letter denotes a non‐significant difference between the values.

Abbreviations: NA, not applicable; SD, standard deviation.

^a^ Repeated measures one‐way analysis of variance.

^b^ Different letters (a, b, c, and d) denote that values were significantly different from each other (*p* < 0.05).

Adherence was not significantly different between weekdays and weekend days (63% vs. 60%, *p* = 0.35), but weight‐bearing activity duration and number of steps/day were higher on weekdays compared to weekend days (3.4 vs. 3.0 h/day, *p* = 0.01; 5782 vs. 5036 steps, *p* = 0.015).

Adherence was lower at home than away from home (59% vs. 74%, *p* < 0.001), activity duration was higher at home than away from home (2.2 vs. 1.2 h/day, *p* < 0.001), and number of steps did not differ significantly between at home and away from home (2866 vs. 2930 steps, *p* = 0.88).

Time spent per daily weight‐bearing activity was statistically significantly different between all activities and ranked from highest‐to‐lowest: standing (2.0 h), walking (1.2 h), shuffling (0.3 h), and stair walking (0.03 h). Adherence levels did not differ between activities (range: 61%–63%, *p* = 0.31).

## 4 DISCUSSION

We investigated adherence to wearing custom‐made footwear in people with diabetes at high risk of ulceration. Average adherence was 63%, varied over the day and was lower at home than away from home. Among multiple demographic, medical, footwear usability, and quality of life factors, no predictor of overall adherence was found. A higher HbA1c level predicted lower adherence at home, and a higher number of daily steps predicted lower adherence away from home, but both models explained a low proportion of the variance (<12%).

Overall adherence to wearing prescribed footwear was 63% and was lower at home (59%) than away from home (74%). This is in line with previous studies [5, 23, 24, 25]. This adherence level is concerning, as it has been associated with higher risk of foot ulceration [2, 26]. We found that participants spent more time in weight‐bearing activity at home than away from home, while the number of steps did not differ. This partly contrasts with previous studies finding the number of steps to be higher at home than away from home [5, 27]. This could reflect that participants in our study may have spent more weight‐bearing time in other activities than walking, such as standing. In any case, the contrast between adherence and activity at home and away from home highlights the need to increase adherence at home. Waaijman et al. [5] conducted a study with a design similar to our study and also found that activity and adherence were highest in the morning and afternoon, lower in the evening, and lowest at the night. Although the activity level is very low at night, the low adherence is concerning. Nighttime activity often consists of visits to the bathroom when people are sleepy, walk barefooted and do not turn on all lights, increasing the risk of trauma to the foot. Activity duration was relatively high during the evening (0.8 h), while adherence declined to 40%. This may be related to people spending most of the evenings at home where adherence is lower. The combination of low footwear adherence and long activity duration at home highlights the need to specifically focus on increasing adherence at home, such as with indoor footwear [28].

Only higher HbA1c was significantly associated with lower adherence at home. This association is likely to reflect some confounding factor, for example, some behavioral factor might be associated with both higher HbA1c and lower footwear adherence at home. It is unclear why this would not apply for adherence away from home; therefore, this should be explored in future studies. Only higher number of daily steps was significantly associated with lower adherence away from home. This indicates that physically more active people were less adherent to their prescribed footwear away from home. This is a surprising result. A reason might be that more active people may engage in more physically and socially diverse contexts compared to less active people, and therefore feel that their prescribed footwear was not appropriate to wear for all contexts. This would fit with the concept of “strategic non‐adherence”, where people balance their quality of life with the costs and benefits of adherence. For example, by refraining from using prescribed footwear in physical and social contexts where the footwear does not “fit” [29]. We suggest that the relation between activity, adherence, and contextual factors should be explored in future research.

One must ask why studies so far, including the current study, have found only few and often inconsistent predictors of footwear adherence [4]. Our study had a similar study design (including most predictors investigated) as two previous studies [5, 7]. When comparing these studies, the adherence predictors found are inconsistent and only explain a low proportion of variance in adherence. This raises questions about the generalizability of the few predictors identified, and—more importantly—raises a need to look for predictors in new areas. The studies so far have focused on variables such as demographic, medical, footwear usability, and quality of life. Other avenues should be included in future research, such as psychological variables, depression, and cognition [4]. Some researchers are now applying structured behavioral models, focusing on capability, opportunity, and motivation to perform diabetic foot self‐management [30]. Such models may open up new avenues for investigating adherence predictors and designing adherence‐improving interventions.

No previous study has separately analyzed predictors for footwear adherence at home and away from home, and several of our results point toward treating adherence at home and away from home as different concepts. First, the absolute adherence levels differed substantially between 59% at home and 74% away from home. Second, different factors predicted adherence at home (HbA1c) and away from home (number of daily steps). Third, the correlation between adherence at home and away from home was moderate (*r* = 0.67). This confirms our approach to separately investigate predictors of adherence at home and away from home, as has also been suggested for offloading devices for ulcer healing [31]. For both research and clinical practice, the results imply that adherence should not be considered as a singular concept but should take contextual and situational factors into account.

A strength of the study was that adherence was defined in terms of all weight‐bearing activities and measured prospectively and objectively with validated methods [21]. A study limitation was that the analyses were conducted as secondary analyses in this prospective cohort study. Thus, no sample size calculation was performed for the current analyses and statistical power was low for some predictors.

## 5 CONCLUSION

In conclusion, we found no predictor of overall adherence to wearing prescribed footwear in people with diabetes at high risk of ulceration. Higher HbA1c predicted lower adherence at home, and higher number of daily steps predicted lower adherence away from home. Our results suggest that footwear adherence at home and away from home should be treated as different concepts in research and clinical practice. Similar to other studies, the identified predictors did not explain much of the variance in adherence and therefore do not substantially improve our understanding of adherence or provide targets for interventions to improve adherence. Future research should shift focus to other potential predictors, for example, psychological variables.

## AUTHOR CONTRIBUTIONS

**Gustav Jarl**: Conceptualization; methodology; formal analysis; writing—original draft; writing—review and editing; visualization. **Chantal M. Hulshof**: Conceptualization; methodology; software; validation; formal analysis; investigation; resources; data curation; writing—review and editing; visualization; project administration. **Kim A. Tijhuis**: software; investigation; data curation; writing—review and editing. **Tessa E. Busch‐Westbroek**: resources; writing—review and editing; funding acquisition. **Sicco A. Bus**: Conceptualization; methodology; validation; resources; writing—review and editing; supervision; funding acquisition. **Jaap J. van Netten**: Conceptualization; methodology; validation; investigation; resources; data curation; writing—review and editing; supervision; project administration; funding acquisition.

## CONFLICT OF INTEREST STATEMENT

Gustav Jarl is a consultant for Novo Nordisk. Chantal M. Hulshof, Kim A. Tijhuis, Tessa E. Busch‐Westbroek, Sicco A. Bus, and Jaap J. van Netten have no potential conflicts of interest to disclose.

## ETHICS STATEMENT

The study conformed to the Declaration of Helsinki. The local medical ethics committee of Amsterdam UMC ruled the study exempt from the requirement for ethical review according to Dutch law under the Medical Research Involving Human Subjects Act in the Netherlands (registration number: W19_429#19.495).

## PATIENT CONSENT STATEMENT

All participants provided written informed consent prior to study participation.

## Data Availability

The data cannot be shared due to patient confidentiality and national laws.
